# Randomized Phase II Study of Docetaxel plus Personalized Peptide Vaccination versus Docetaxel plus Placebo for Patients with Previously Treated Advanced Wild Type EGFR Non-Small-Cell Lung Cancer

**DOI:** 10.1155/2016/1745108

**Published:** 2016-05-04

**Authors:** Koichi Takayama, Shunichi Sugawara, Yasuo Saijo, Makoto Maemondo, Atsushi Sato, Shinzo Takamori, Taishi Harada, Tetsuro Sasada, Tatsuyuki Kakuma, Junji Kishimoto, Akira Yamada, Masanori Noguchi, Kyogo Itoh, Yoichi Nakanishi

**Affiliations:** ^1^Department of Respirology, Kyoto Prefectural University of Medicine, Kyoto, Japan; ^2^Sendai Kousei Hospital, Sendai, Japan; ^3^Department of Medical Oncology, Niigata University Graduate School of Medical and Dental Sciences, Niigata, Japan; ^4^Department of Respiratory Medicine, Miyagi Cancer Center, Miyagi, Japan; ^5^Department of Medical Oncology, Hirosaki University Graduate School of Medicine, Hirosaki, Japan; ^6^Department of Surgery, Kurume University School of Medicine, Kurume, Japan; ^7^Research Institute for Diseases of the Chest, Graduate School of Medical Sciences, Kyushu University, Fukuoka, Japan; ^8^Kanagawa Cancer Center Research Institute, Yokohama, Japan; ^9^Biostatistics Center, School of Medicine, Kurume University, Kurume, Japan; ^10^Center for Clinical and Translational Research, Kyushu University, Fukuoka, Japan; ^11^Cancer Vaccine Development Division, Kurume University Research Center for Innovative Cancer Therapy, Kurume University School of Medicine, Kurume, Japan; ^12^Division of Clinical Research, Research Center for Innovative Cancer Therapy, Kurume University School of Medicine, Kurume, Japan; ^13^Cancer Vaccine Center, Kurume University School of Medicine, Kurume, Japan

## Abstract

*Objectives*. To evaluate the efficacy and safety of personalized peptide vaccination (PPV) combined with chemotherapy for patients with previously treated advanced non-small-cell lung cancer (NSCLC).* Patients and Methods*. Previously treated PS0-1 patients with IIIB/IV EGFR (epidermal growth factor receptor) wild genotype NSCLC were randomly assigned to docetaxel (60 mg/m^2^ on Day 1) plus PPV based on preexisting host immunity or docetaxel plus placebo. Docetaxel administration was repeated every 3 weeks until disease progression. Personalized peptides or placebo was injected subcutaneously weekly in the first 8 weeks and biweekly in subsequent 16 weeks. The primary efficacy endpoint was progression-free survival (PFS).* Results*. PPV related toxicity was grade 2 or less skin reaction. The median PFS for placebo arm and PPV arm was 52 days and 59 days, respectively. There was no significant difference between two arms by log-rank test (*p* = 0.42). Interestingly, PFS and overall survival (OS) in humoral immunological responder were significantly longer than those in nonresponder.* Conclusion*. PPV did not improve the survival in combination with docetaxel for previously treated advanced NSCLC. However, PPV may be efficacious for the humoral immunological responders and a further clinical investigation is needed.

## 1. Introduction

Lung cancer is a leading cause of cancer-related death in Japan. Although the treatment with platinum-based chemotherapy improves survival and quality of life (QOL) in patients with advanced non-small-cell lung cancer (NSCLC), a substantial population of patients progress and should be offered second-line treatment [1]. With unsurpassed efficacy compared with other chemotherapeutic regimens, docetaxel alone is the current standard as second-line chemotherapy for advanced NSCLC [2].

Itoh and Yamada developed a new approach of personalized peptide vaccination (PPV), in which vaccine antigens are selected and administered based on preexisting host immunity before vaccination [3], and have shown promising results of PPV in various types of advanced cancers [4–6]. With similar PPV treatment, Yoshiyama et al. conducted a phase II trial in refractory NSCLC patients recently [7], and forty-one patients were enrolled. Median overall survival (OS) was 304 days with a one-year survival rate of 42%. Most frequent toxicity of PPV was skin reaction at the injection sites, but no serious adverse events were observed. In this study, we conducted a multicenter, randomized, phase II trial to evaluate whether the combination regimen of docetaxel plus PPV provides better progression-free survival than docetaxel alone in patients with previously treated NSCLC.

## 2. Patients and Methods

### 2.1. Patients

Patients aged 20 years or older with histologically or cytologically confirmed inoperable stage IIIB/IV or recurrent NSCLC with epidermal growth factor receptor (EGFR) wild genotype were eligible for enrollment. Other eligibility criteria included measurable disease, ECOG (Eastern Cooperative Oncology Group) PS (performance status) of 0-1, life expectancy of at least 12 weeks, and adequate hematologic (hemoglobin >8.0 g/dL, neutrophil count >2,000/*μ*L, lymphocyte count >1,200/*μ*L, and platelet count >100,000/*μ*L), hepatic (bilirubin <1.5 mg/dL, aspartate transaminase/alanine transaminase <2.5x upper limit of normal), and renal (serum creatinine >1.5 mg/dL) function. Patients who received prior one or two chemotherapy regimens were eligible. Prior radiation therapy or chemotherapy was to be completed at least 4 weeks before enrollment, and the patient was required to be completely recovered from all adverse effects due to prior treatment. All patients must be of positive status for human leukocyte antigen- (HLA-) A2, HLA-A24, HLA-A26, or HLA-A3 supertype (HLA-A3, HLA-A11, HLA-A31, and HLA-A33) and have at least two or more peptide-specific IgGs in the preregistration immunological screening. Patients with active serious infection or other serious underlying medical conditions were ineligible. Immunocompromised patient or the patient with systemic administration of corticosteroid was excluded. The study protocol was approved by the institutional review boards with jurisdiction over the sites where patients were registered for the study and registered in the UMIN Clinical Trials Registry (UMIN number 000003521). All patients provided informed consent before enrollment.

### 2.2. Treatment Plan

On enrollment, patients were randomly assigned to either a docetaxel 60 mg/m^2^ as a 60 min intravenous infusion on Day 1 plus placebo injection or docetaxel plus PPV. Placebo or PPV was administered subcutaneously as scheduled, described below. Chemotherapy was repeated every 3 weeks until disease progression. In this study, 31 peptides whose safety and immunological effects had been confirmed in previously conducted clinical studies were employed for vaccination (12 peptides for HLA-A2, 14 peptides for HLA-A24, 9 peptides for HLA-A3 supertype, and 4 peptides for HLA-A26 as shown in supplementary Table  1 in Supplementary Material available online at http://dx.doi.org/10.1155/2016/1745108). All peptides were prepared under conditions of Good Manufacturing Practice by the PolyPeptide Laboratories (San Diego, CA) and American Peptide Company (Vista, CA). Each of the selected peptides was mixed with incomplete Freund's adjuvant (Montanide ISA51VG; Seppic, Paris, France) and emulsified in the 5 mL plastic syringe. Freund's adjuvant with saline without any peptide was used as placebo. Based on the results of specific IgG titer in patients plasma, two to four peptides were selected for vaccination in PPV arm. PPV or placebo was injected subcutaneously weekly in the first 8 weeks and biweekly in subsequent 16 weeks after 1st cycle of docetaxel administration. The study was terminated if the patients required more dose reduction of docetaxel than 50 mg/m^2^ or the delay was longer than two weeks of docetaxel administration.

### 2.3. Baseline and Follow-Up Assessments

Pretreatment evaluation included HLA typing and immunological tests as described below in detail, except common blood test and chest imaging studies. Adverse events were monitored according to the National Cancer Institute Common Terminology Criteria for Adverse Events version 3.0 (CTCAE Ver. 3). All patients were assessed for response by computed tomography scans after every two cycles of chemotherapy. The PFS was calculated from the day of randomization until the day of the first evidence of disease progression or death. OS was measured from the day of randomization to death.

### 2.4. Measurement of Humoral and T-Cell Responses Specific to the Vaccinated Peptides

To study the humoral immunological response, peptide-specific IgG levels were measured by a Luminex system (Luminex, Austin, TX), as reported previously [8]. It was defined to be positive IgG response if the total titers of selected peptide-specific IgG increased 2-fold higher than those in the pretreatment plasma. For the cellular immunological response, T-cell responses specific to the vaccinated peptides were evaluated by interferon-g enzyme-linked immunospot (ELISPOT) assay using peripheral blood mononuclear cells (PBMCs), as reported previously [7]. Peptide-specific T-cell responses were evaluated by the difference between the numbers of spots per 10^5^x PBMCs in response to the vaccine peptides. It was also defined to be positive cellular immunological response if the total number of spots is 2-fold higher than those in the prevaccination. These immunological assays were performed before randomization for a baseline data, after 8 and 16 injections of placebo or PPV and at the treatment termination.

### 2.5. Statistical Analysis

Efficacy analyses were completed for the intent-to-treat population. Safety analyses were performed for the population who received at least one dose of docetaxel after random assignment. The primary efficacy endpoint was PFS. The secondary efficacy endpoints were overall response rate (ORR), disease control rate (DCR), and immunological response specific to the vaccinated peptides. The present study was designed to detect 2-month prolongation of PFS in docetaxel plus PPV arm. To attain an 80% power at a one-sided significance level of 0.05, assuming PFS of docetaxel plus placebo arm as 2 months [9], 64 patients (32 per each arm) were required with 10% dropout rate. Both of PFS and OS were estimated with the Kaplan-Meyer method. The comparisons of OS and PFS between arms were assessed by the log-rank test. Hazard ratio was calculated with Cox proportional hazard model. The difference of the ORR or DCR was analyzed by Fisher's exact test. The exploratory analysis of subgroups by immunological response was performed with similar methods as described above. All analyses were carried out with R 2.15.1 software.

## 3. Results

### 3.1. Patient Characteristics

Between January 2010 and September 2013, 67 patients were enrolled onto the study at 5 sites. After excluding 17 ineligible cases because of smaller number of lymphocytes, mutant* EGFR* genotype, smaller number of neutrophils, and others, remaining 50 patients were randomly assigned to docetaxel plus placebo (placebo arm, *n* = 24) or docetaxel plus PPV (PPV arm, *n* = 26). Baseline characteristics of patients are summarized in Table 1 and were well balanced in two treatment arms in terms of age, gender, PS, clinical stage, and histology.

### 3.2. Treatment Delivery

In the placebo arm, the median number of docetaxel times of administration and placebo injections was 3 cycles and 8 injections, respectively. In the PPV arm, the median number of docetaxel times of administration and personalized peptide injections was 3 cycles and 8 injections, respectively. There was no statistical difference in each drug delivery between two treatment arms. Three patients (12.5%) in placebo arm and four patients (15.4%) in PPV arm completed the 16-time placebo or personalized peptide injections as scheduled in the protocol.

### 3.3. Immunological Analysis

Out of sixty-seven patients enrolled in this study, sixty-six patients were applied for the immunological screening for thirty-one candidate peptides. All patients met the HLA typing criteria and each of HLA-A2, HLA-A24, HLA-A26, or HLA-A3 superfamily was positive. Sixty-five patients (98.5%) showed the presence of two or more peptide-specific IgGs in the serum, two IgGs (6.1%), three IgGs (10.6%), and four or more IgGs (80.3%). Only one patient (1.5%) did not show increase of any peptide-specific IgG. The peptides corresponding to frequently detected (>10%) peptide-specific IgG were as follows: Lck-488 (46.2%), SART2-93 (40.0%), PAP-213 (32.3%), PSA-248 (27.7%), PTHrP-102 (24.6%), Lck-486 (24.6%), CypB-129 (24.6%), Lck-208 (23.1%), WHSC2-141 (12.3%), SART3-511 (10.8%), and SART3-309 (10.8%). In the PPV arm (*n* = 26), one patient failed to examine the immunological response after vaccination and remaining 25 patients were analyzed after 8 and/or 16 vaccinations and study termination. According to our previous clinical results, the patient who showed 2-fold or higher increase of total titer for selected peptides at any examination point was defined as the humoral immunological responder. In the PPV arm, 14 patients were categorized into humoral responder. Likewise, the patient who showed 2-fold or more increase of total number of spots for selected peptides in ELISPOT assay was defined as the cellular immunological responder. In the PPV arm, 14 patients were categorized into cellular responder. Nine patients showed both humoral and cellular immunological responses positively. In the placebo arm, two patients failed to examine the immunological response after vaccination and remaining 22 patients were analyzed. Only one patient showed the 2-fold or more increase of peptide-specific IgGs in the serum after 8 and 16 injections of placebo. Skin reaction is a possible physical manifestation reflecting immunological response to PPV or placebo. Each number of the patients with skin reaction in placebo arm or PPV arm was four or thirteen patients, respectively. The results of immunological analysis are summarized in Table 2.

### 3.4. Safety

Generally the profile of adverse events was similar to the report in previous clinical trial [9]. Most frequent toxicity was neutrocytopenia mainly due to docetaxel administration. As nonhematological grade 3 (G3) toxicities, appetite loss, neuropathy, active pulmonary infection, and interstitial lung disease (ILD) were reported. Any new safety signal was not detected comparing with previous clinical studies. There was no statistical difference in toxicities between placebo arm and PPV arm.  Table 3 provides a summary of G3 or more toxicities. Concerning the injection related adverse events, four patients (16.7%) in placebo arm and 13 patients (50%) in PPV arm claimed G1 or G2 skin reaction at injection site. The frequency was higher in PPV arm significantly. The possible explanation was the immunological reaction against injected peptide. In the 13 patients with positive skin reaction, 9 patients (69%) showed the increase of peptide-specific IgG as a positive response, while, in the 12 patients with negative skin reaction, only 5 patients (42%) showed the increase of IgG.

### 3.5. Efficacy

**Figure 1:**
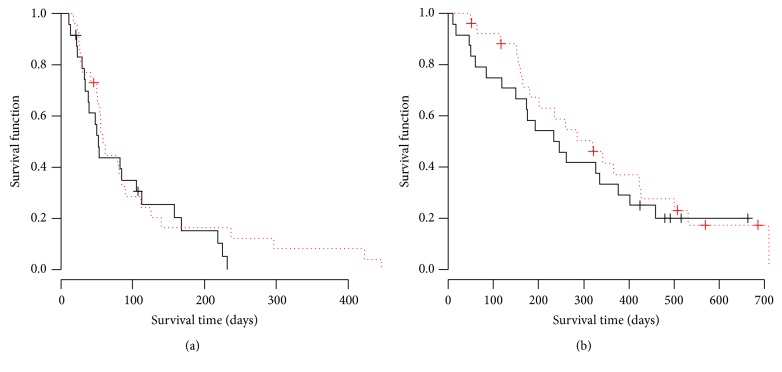
Survival curves of placebo and PPV arm. (a) Solid and dotted lines indicate the PFS curve by Kaplan-Meyer method in placebo and PPV arms, respectively. No significant difference was noted between both arms (*p* = 0.42, log-rank test). The median PFS in the placebo and PPV arm was 52 days and 59 days, respectively. Hazard ratio of PFS curve in PPV arm was 0.78 (95% CI 0.43–1.42). (b) Solid and dotted lines indicate the OS curve by Kaplan-Meyer method in placebo and PPV arms, respectively. No significant difference was noted between both arms (*p* = 0.49, log-rank test). The median PFS in the placebo and PPV arm was 233 days and 320 days, respectively. Hazard ratio of OS curve in PPV arm was 0.80 (95% CI 0.42–1.51).

**Figure 2:**
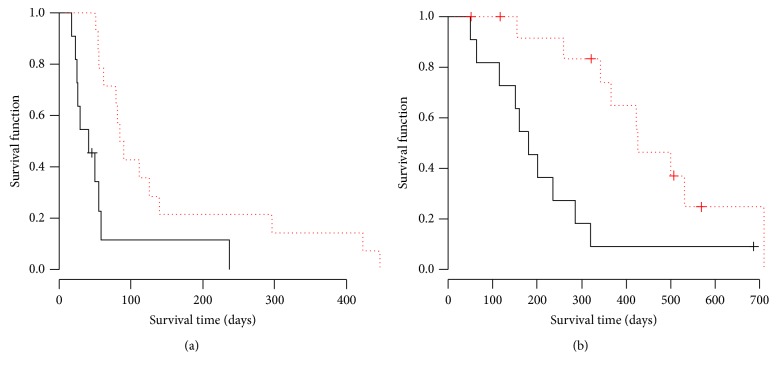
Survival curves of immunological responder and nonresponder in PPV arm. (a) Solid and dotted lines indicate the PFS curve by Kaplan-Meyer method in humoral immunological nonresponder and responder group of PPV arm, respectively. There was a significant difference between both arms (*p* = 0.0034, log-rank test). The median PFS in the nonresponder and responder was 41 days and 84 days, respectively. Hazard ratio of PFS curve in responder group was 0.28 (95% CI 0.11–0.69). (b) Solid and dotted lines indicate the OS curve by Kaplan-Meyer method in humoral immunological nonresponder and responder group of PPV arm, respectively. There was a significant difference between both arms (*p* = 0.0049, log-rank test). The median OS in the nonresponder and responder group was 181 days and 427 days, respectively. Hazard ratio of OS curve in responder group was 0.27 (95% CI 0.10–0.71).

**Figure 3:**
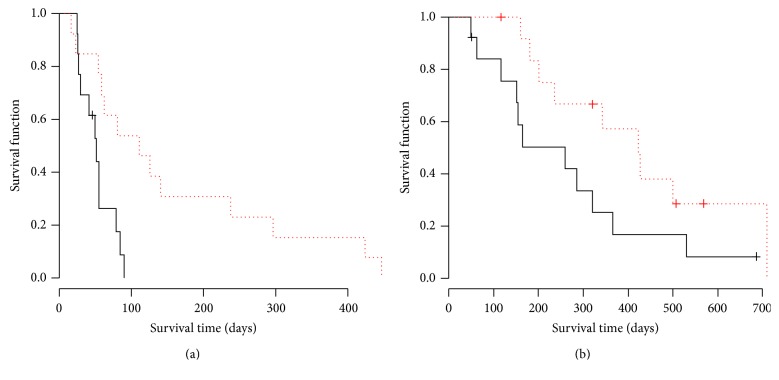
Survival curves of the patients group with or without skin reaction in PPV arm. Dotted and solid lines indicate the PFS or OS curve by Kaplan-Meyer method of the patients group with or without skin reaction in PPV arm, respectively. (a) PFS curve. There was a significant difference between both arms (*p* = 0.005, log-rank test). The median PFS in the patients group with or without skin reaction was 51 days and 111 days, respectively. Hazard ratio of PFS curve in skin reaction group was 0.25 (95% CI 0.09–0.70). (b) OS curve. There was a marginal but not significant difference between both arms (*p* = 0.073, log-rank test). The median OS in the patients group with or without skin reaction was 259 days and 423 days, respectively. Hazard ratio of OS curve in the skin reaction group was 0.44 (95% CI 0.18–1.11).

There was no complete response (CR) patient in both arms. The ORR for placebo and PPV arm was 8.3% and 3.8%, respectively. The DCR for placebo and PPV arm was 20.8% and 11.5%, respectively. There was no significant difference in ORR (*p* = 0.60) and DCR (*p* = 0.46) between two arms. PFS and OS Kaplan-Meyer curves were shown in Figures 1(a) and 1(b). The median PFS for placebo and PPV was 53 days and 59 days, respectively. There was no significant difference between two arms by log-rank test (*p* = 0.42). Hazard ratio of PFS curve in PPV arm was 0.78 (95% CI 0.43–1.42). The median OS for placebo and PPV was 233 days and 320 days, respectively. There was no significant difference between two arms by log-rank test (*p* = 0.49). Hazard ratio of OS curve in PPV arm was 0.80 (95% CI 0.42–1.51). Based on the results, we concluded that the addition of PPV on docetaxel treatment did not improve tumor response and survival for wild type* EGFR* advanced NSCLC as a second- or third-line setting. In the next step, we examined the survival time in immunological responder and nonresponder as an ad hoc analysis. Interestingly, PFS in humoral immunological responder was significantly longer than that in nonresponder with a hazard ratio (HR) 0.28 (*p* < 0.01) as shown in Figure 2(a). OS in the responder was also significantly longer than that in nonresponder with HR 0.27 (*p* < 0.01) as shown in Figure 2(b). The cellular immunological responder did not show the improvement in PFS or OS. Since the skin reaction is thought to reflect the immunological response to injected peptide, we also examined the survival time in the patient group with or without skin reaction. In the PPV arm, the patients with G1 or G2 skin reaction (*n* = 13) showed the significant improvement of PFS (HR 0.25, *p* < 0.01) and marginal improvement of OS (HR 0.44, *p* = 0.073) compared with those in the patients without skin reaction as shown in Figures 3(a) and 3(b), while, in the placebo arm, the patients with skin reaction (*n* = 4) did not show any survival improvement. Hazard ratio in PFS and OS was 0.94 (*p* = 0.92) and 0.75 (*p* = 0.65), respectively.

## 4. Discussion

Three phase III clinical trials for previously treated EGFR unselected advanced NSCLC, V-15-32 [9], JCOG0104 [10], and DELTA [11] were conducted recently in Japan. In these trials, docetaxel monotherapy was used in the control arm. In the efficacy of docetaxel monotherapy, these phase III trials reported 2.0–2.9 months of PFS and 10.1–14.0 months of OS. Comparing with these historical control data, the results of efficacy in this study were warranted considering that target population is limited to* EGFR* wild type and the study includes third-line setting.

As a new strategy for cancer treatment, there have recently been noteworthy advances in the clinical application of cancer immunotherapy [12]. In the field of lung cancer, recent reports showed the certain clinical efficacy of lung cancer with programmed cell death-1 (PD-1) signal blockade with monoclonal anti-PD-1 antibody as well as ipilimumab [13]. Actually, it was reported that nivolumab, monoclonal anti-PD-1 antibody, significantly improves the overall survival for the patients with advanced, previously treated squamous-cell NSCLC compared with docetaxel in a phase III trial recently [14]. Augmented immune response against lung cancer cells is expected to improve the prognosis of advanced lung cancer patients. In combination with these immune checkpoint inhibitors, cancer vaccination strategy may be enhanced.

In this regard, we have developed a personalized peptide vaccination as a novel immunotherapeutic approach for various kinds of cancer [15]. Since immune cell repertoires are quite diverse and heterogeneous, antitumor immunity might be substantially different among individuals. In our personalized cancer vaccination, appropriate peptide antigens for vaccination are screened and selected from a list of vaccine candidates in each patient, based on preexisting host immunity. Unfortunately, current study could not improve PFS and OS for previously treated unselected EGFR wild type patients in addition to docetaxel monotherapy as a standard regimen. The negative result is partly due to the considerable numbers of immunological nonresponders included in the trial. In the ad hoc analysis, PPV in addition to docetaxel significantly improves PFS and OS in humoral immunological responder group defined as twofold or higher peptide-specific IgG compared with nonresponder in PPV arm. Therefore, if the humoral immunological responder is selected, PPV may be confirmed to be beneficial in previously treated advanced NSCLC. To select the immunological responder, we need to search predictive biomarkers. Another postvaccination predictor candidate is a skin reaction as a result of delayed hypersensitivity local response to injected peptide. As shown in Figure 3, the patient group with skin reaction showed the better survival compared with those without skin reaction.

## 5. Conclusion

In conclusion, primary endpoint was not met in this clinical trial and PPV did not improve the survival in combination with docetaxel for previously treated EGFR wild type advanced NSCLC. However, PPV may be efficacious for the humoral immunological responders and a further clinical investigation is needed to select the patients benefitted by cancer vaccine appropriately.

## Supplementary Material

List of peptides for vaccination.

## Figures and Tables

**Table 1:** Patients characteristics.

	Docetaxel + placebo	Docetaxel + PPV
*N*	24	26
Age (median)	66	62.5
Gender (male/female)	22/2	19/7
PS (0/1)	8/16	3/23
c-stage (IIIB/IV/recurrent)	4/16/4	7/18/1
Histology (Ad/non-Ad)	21/3	18/8

PS: performance status and Ad: adenocarcinoma.

**Table 2:** Immunological response.

	Docetaxel + placebo	Docetaxel + PPV
Prevaccination		
*N*	24	26
2 peptide-specific IgGs	1	3
3 peptide-specific IgGs	1	4
4 peptide-specific IgGs	22	19
Postvaccination		
*N*	22	25
Humoral responder	1	14
Cellular responder	Not examined	14
Positive skin reaction	4	13

**Table 3:** Hematological and nonhematological toxicities.

	AE (G3/4)	Docetaxel + placebo	Docetaxel + PPV
Hematologic	Leukocytopenia	33.3%	46.2%
	Neutrocytopenia	100.0%	76.9%
	Lymphocytopenia	4.2%	0%
	FN	4.2%	3.8%

Nonhematologic	Appetite loss	4.2%	0%
	Neuropathy	4.2%	0%
	Infection	4.2%	0%
	ILD	4.2%	7.7%

Injection related	Skin reaction (G1/2)	16.7%	50.0%

FN, febrile neutrocytopenia; ILD, interstitial lung disease.
