# Supplementary material for: Randomized Phase II Study of Docetaxel plus Personalized Peptide Vaccination versus Docetaxel plus Placebo for Patients with Previously Treated Advanced Wild Type EGFR Non-Small-Cell Lung Cancer
Source: J Immunol Res. 2016 May 4;2016:1745108. doi: 10.1155/2016/1745108 (PMC4870343; doi:10.1155/2016/1745108)
Supplement: Supplementary file 1 — List of peptides for vaccination. [file 1745108.f1.docx]

Supplementary Table 1 Peptide candidates used for personalized peptide vaccines

| Original protein | Peptide name | Position of  peptide | Amino acid  sequence | HLA  Restriction |
| --- | --- | --- | --- | --- |
| Cyclophilin B  p56 lck  p56 lck  ppMAPkkk  WHSC2  HNRPL  UBE2V  UBE2V  WHSC2  HNRPL  SART3  SART3  SART2  SART3  p56 lck  PAP  PSA  EGF-R  MRP3  MRP3  SART2  p56 lck  p56 lck  PSMA  EZH2  PTHrP  SART3  SART3  p56 lck  p56 lck  PAP | CypB-129  Lck-246  Lck-422  MAP-432  WHSC2-103  HNRPL-501  UBE-43  UBE-85  WHSC2-141  HNRPL-140  SART3-302  SART3-309  SART2-93  SART3-109  Lck-208  PAP-213  PSA-248  EGFR-800  MRP3-503  MRP3-1293  SART2-161  Lck-486  Lck-488  PSMA-624  EZH2-735  PTHrP-102  SART3-511  SART3-734  Lck-90  Lck-449  PAP-248 | 129-138  246-254  422-430  432-440  103-111  501-510  43-51  85-93  141-149  140-148  302-310  309-317  93-101  109-118  208-216  213-221  248-257  800-809  503-511  1293-1302  161-169  486-494  488-497  624-632  735-743  102-111  511-519  734-742  90-99  449-458  248-257 | KLKHYGPGWV  KLVERLGAA  DVWSFGILL  DLLSHAFFA  ASLDSDPWV  NVLHFFNAPL  RLQEWCSVI  LIADFLSGL  ILGELREKV  ALVEFEDVL  LLQAEAPRL  RLAEYQAYI  DYSARWNEI  VYDYNCHVDL  HYTNASDGL  LYCESVHNF  HYRKWIKDTI  DYVREHKDNI  LYAWEPSFL  NYSVRYRPGL  AYDFLYNYL  TFDYLRSVL  DYLRSVLEDF  TYSVSFDSL  KYVGIEREM  RYLTQETNKV  WLEYYNLER  QIRPIFSNR  ILEQSGEWWK  VIQNLERGYR  GIHKQKEKSR | A2，A3sup  A2  A2，A3sup  A2，A26  A2，A3sup，A26  A2，A26  A2  A2  A2  A2  A2  A2  A24  A24，A3sup，A26  A24  A24  A24  A24  A24  A24  A24  A24  A24  A24  A24  A24  A3sup  A3sup  A3sup  A3sup  A3sup |

A3sup: HLA-A3 supertype (A3, A11, A31, or A33); EGF-R: Epidermal Growth Factor Receptor; EZH2: enhancer of zeste homolog 2; HNRPL: heterogeneous nuclear ribonucleoprotein L; ppMAPkkk: partial putative mitogen-activated protein kinase kinase kinase; MRP3:multidrug resistance-associated protein 3; PAP: Prostatic acid phosphatase; PSA: prostate specific antigen; PSMA: Prostate specific membrane antigen; PTHrP :parathyroid hormone- related peptide; SART2: squamous cell carcinoma antigen recognized by T cells 2; SART3: squamous cell carcinoma antigen recognized by T cells 3; UBE2V: ubiquitin-conjugated enzyme variant Kua; WHSC2: Wolf-Hirschhorn syndrome candidate 2.
